# Health-Promoting Properties of Sorghum Bioactive Compounds—A Comprehensive Bibliometric Analysis

**DOI:** 10.3390/nu17233732

**Published:** 2025-11-28

**Authors:** Jakub Frankowski, Aleksandra Zielińska, Mateusz Labudda, Maciej Ireneusz Kluz, Miroslava Kačániová, Przemysław Łukasz Kowalczewski

**Affiliations:** 1 School of Medical and Health Sciences, VIZJA University, Okopowa 59, 01-043 Warszawa, Poland; j.frankowski@vizja.pl (J.F.);; 2 Department of Pharmacology and Phytochemistry, Institute of Natural Fibres & Medicinal Plants, National Research Institute, Kolejowa 2, 62-064 Plewiska, Poland; 3 Department of Biochemistry and Microbiology, Institute of Biology, Warsaw University of Life Sciences-SGGW, Nowoursynowska 159, 02-776 Warsaw, Poland; mateusz_labudda@sggw.edu.pl; 4 Collegium Medicum, Andrzej Frycz Modrzewski Krakow University, Gustawa Herlinga-Grudzińskiego 1, 30-705 Kraków, Poland; 5 Institute of Horticulture, Faculty of Horticulture and Landscape Engineering, Slovak University of Agriculture, Tr. A. Hlinku 2, 94976 Nitra, Slovakia

**Keywords:** antioxidants, natural compounds, oxidative stress, glycemic index, cardiovascular health, metabolic disorders

## Abstract

**Background/Objectives**: Sorghum (*Sorghum bicolor* Moench) is a globally cultivated cereal and an increasingly important crop in Europe due to its exceptional adaptability to climate change and low input requirements. It represents a rich source of bioactive compounds, including phenolic acids, flavonoids, tannins, and 3-deoxyanthocyanidins, which exhibit strong antioxidant, anti-inflammatory, and metabolic regulatory properties. **Methods**: This review, as a bibliometric analysis, summarizes and discusses current evidence on the health-promoting effects of sorghum, with particular emphasis on its antioxidant, metabolic, and cardiovascular benefits. **Results**: The antioxidant potential of sorghum is mainly attributed to its phenolic profile, which varies considerably depending on genotype, environmental factors, and processing methods. Human and animal studies demonstrate that sorghum-derived polyphenols enhance endogenous antioxidant enzyme activity, decrease oxidative stress biomarkers, and beneficially modulate gut microbiota composition. Sorghum-based foods are characterized by low to medium glycemic indices, promoting improved postprandial glucose regulation, insulin sensitivity, and prolonged satiety. Furthermore, sorghum bioactive peptides and phenolic acids contribute to cardiovascular health by lowering serum cholesterol levels, reducing inflammatory markers, and exhibiting angiotensin-converting enzyme (ACE) inhibitory activity. **Conclusions**: Overall, sorghum constitutes a gluten-free, antioxidant-rich grain with significant potential for mitigating oxidative stress, supporting metabolic balance, and preventing chronic non-communicable diseases.

## 1. Introduction

Sorghum (*Sorghum bicolor* Moench) is one of the most important cereal crops globally, serving as a staple food for millions of people, particularly in semi-arid and arid regions. In recent years, sorghum has gained increasing scientific attention also in Europe—not only as a resilient crop with high environmental adaptability but also as a valuable source of bioactive compounds with promising health-promoting potential. Unlike other major cereals such as wheat, rice, or maize, sorghum contains a unique spectrum of phytochemicals, including phenolic acids, flavonoids, tannins, and 3-deoxyanthocyanidins, which contribute to its antioxidant, anti-inflammatory, and metabolic regulatory effects [1,2,3,4,5,6].

A growing body of evidence suggests that these bioactive constituents play a significant role in the prevention and management of chronic non-communicable diseases, such as type 2 diabetes, obesity, cardiovascular disorders, and certain cancers [7,8,9]. The mechanisms underlying these effects are associated with the modulation of oxidative stress, regulation of inflammatory and apoptotic pathways, and beneficial modulation of gut microbiota composition, all of which are closely linked to the pathophysiology of these disorders. Furthermore, sorghum’s gluten-free nature makes it an attractive alternative for individuals with celiac disease or gluten sensitivity, extending its therapeutic relevance beyond basic nutrition [10,11,12,13].

Despite increasing recognition of sorghum’s nutritional and functional potential, its bioactive components remain underexplored compared with those of other cereals. The diversity in sorghum genotypes, processing techniques, and cultivation conditions strongly affects the concentration, composition, and bioavailability of its phytochemicals, which may influence their biological activity in vivo. Importantly, recent pharmacological studies have revealed that sorghum-derived polyphenols can attenuate oxidative damage in tissues, modulate lipid metabolism, and protect endothelial function, indicating their potential as natural agents for disease prevention and adjunctive therapy [10,14,15,16].

Therefore, this review, as a bibliometric analysis, aims to summarize and critically evaluate the current knowledge on the bioactive compounds present in sorghum and their health-promoting properties from a biomedical perspective, with emphasis on their antioxidant, anti-inflammatory, metabolic, and cardiovascular effects. The discussion focuses on molecular and physiological mechanisms through which sorghum-derived phytochemicals may contribute to maintaining cellular homeostasis, reducing chronic inflammation, and supporting the prevention of non-communicable diseases. The connection between the two principal sections of the manuscript lies in their complementary functions. The narrative review summarizes the existing evidence on the health-promoting properties of sorghum bioactive compounds, while the bibliometric analysis tracks global research trends and thematic directions within this field. Collectively, they offer both a scientific overview and a contextual perspective on research, illuminating where current knowledge is concentrated and identifying significant gaps that remain.

## 2. Materials and Methods

### 2.1. Literature Search Methodology

To collect relevant literature, a narrative review approach was applied, combining a structured database search with critical content analysis. The literature search was carried out between September and November 2025 across major scientific databases, including Web of Science, Scopus, ScienceDirect, Google Scholar, and PubMed. The primary search terms comprised “sorghum,” “sorghum seeds,” “bioactive compounds,” “health-promoting properties,” “antioxidants,” “analysis,” “oxidative stress,” “glycemic index,” “cardiovascular health,” “metabolic disorders,” and related keywords.

The inclusion criteria were restricted to peer-reviewed research articles published in English. Non-scientific sources such as popular science materials, dissertations, and editorials were excluded to maintain a high standard of scientific rigor and relevance. Following the initial screening, a detailed analysis was conducted to prioritize studies offering empirical data, theoretical insights, or substantial contributions to the fields of food science, nutrition, and the dietary applications of sorghum seeds.

Although this review does not strictly adhere to systematic review methodology, the adopted strategy ensured a broad and representative synthesis of the current knowledge base, emphasizing the nutritional and functional potential of sorghum seeds for specific population groups. The scope of the research was limited to sorghum cultivated in Europe. Given the accelerating effects of climate change, sorghum has become an increasingly important crop on this continent. Together with active breeding initiatives, this trend highlights the substantial potential of sorghum and the need for further research in this area. Accordingly, the chosen topic, geographic focus, and time frame were selected to establish a comprehensive foundation for future investigations.

### 2.2. Bibliometric Analysis of Sorghum in Medicine and Healthcare

A bibliometric analysis was conducted to explore the global research landscape linking sorghum with its applications in medicine and healthcare. The dataset was retrieved from the Scopus database. No temporal or language restrictions were imposed to capture the entire research output associated with the topic. The resulting records were exported in a compatible format and analyzed using VOSviewer (version 1.6.19), a software widely used for constructing and visualizing bibliometric networks [17,18].

Three types of analyses were performed:

1. Co-citation analysis—identifying patterns of frequently cited publications to reveal intellectual linkages within the field.
2. Co-authorship by country—mapping international collaboration networks among contributing nations.
3. Keyword co-occurrence analysis—detecting thematic clusters of research activity based on author-provided and indexed keywords.

For all analyses, a threshold of one (1) was applied, meaning that every element (publication, author, or country) appearing at least once in the dataset was included. This inclusive approach aimed to capture both dominant research actors and emerging contributors. Fractional counting was used to normalize contributions among multi-country and multi-author publications. Network maps were generated using the association strength normalization method, and visualization parameters (node size, link strength, and color clustering) were automatically scaled by VOSviewer.

## 3. Results of Literature Investigation

### 3.1. Antioxidant Effects

Antioxidant activity in sorghum cultivated in Europe and other places has emerged as a significant area of nutritional and biochemical research, particularly in relation to its role in preventing chronic diseases. The antioxidant properties of sorghum are primarily attributed to the concentrations of phenolic acids and flavonoids present in the grain. Distinct cultivars, including red, black, and white sorghum, display unique bioactive profiles [19,20].

**Figure 1:**
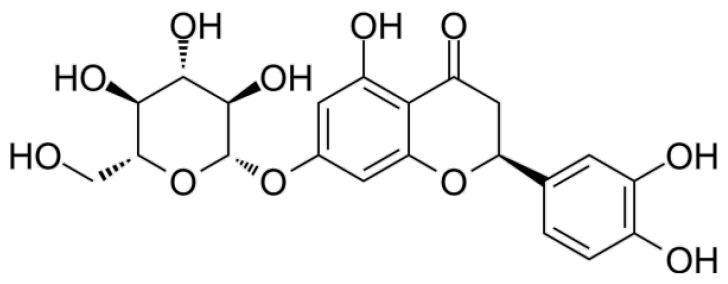
Eriodictyol linked to a sugar moiety of the hexose type.

Red sorghum is notably high in eriodictyol-O-hexoside (Figure 1), which corresponds to its elevated Trolox equivalent antioxidant capacity (TEAC), thereby indicating substantial antioxidant activity. In vitro research indicates that red cultivars, with high concentrations of this compound, correlate with strong antioxidant activity [21].

Conversely, black sorghum is characterized by specific pyrano-flavonoid derivatives that enhance its radical scavenging capacity. White sorghum, while exhibiting the lowest radical scavenging activity, is rich in phenolamides and flavones, such as apigenin-7-O-hexoside. These findings suggest that specific sorghum varieties should be considered for integration into functional food products based on their genotype. Furthermore, the variability among genotypes underscores the need to incorporate sorghum breeding programs that aim to optimize health benefits related to oxidative stress in experimental conditions. This genotype-dependent variability further underscores the potential of sorghum as a valuable dietary option for personalized dietary recommendations, both at the individual and population levels [21,22].

Recent studies have revealed that the bran of brown tannin sorghum varieties offers a significantly richer source of polyphenols compared to whole grain sorghum, with levels surpassing those of whole grain by a factor of six. This finding correlates with the higher antioxidant activity observed in brown tannin sorghum bran, which exhibits 3.5 times the antioxidant potential of whole grain sorghum. 2,2-diphenyl-1-picrylhydrazyl (DPPH) and related assays further substantiate the superior antioxidant activity of sorghum bran [23,24].

In animal model studies, dietary supplementation with sorghum bran has been associated with notable biological responses, including increased activity of endogenous antioxidant enzymes such as superoxide dismutase (SOD) and catalase, alongside reduced levels of oxidative stress biomarkers, including malondialdehyde. Additionally, advanced extraction methods, such as subcritical water extraction, have yielded elevated levels of extracted antioxidants. Collectively, these findings suggest that sorghum bran extracts may effectively inhibit the proliferation of HepG2 cells (epithelial-like cells derived from a male patient with hepatocellular carcinoma), highlighting their potential application in mitigating oxidative stress and preventing chronic diseases [24].

Numerous human intervention studies have investigated the antioxidant potential of bioactive compounds present in sorghum. In a randomized, controlled crossover study, participants who consumed red sorghum pasta demonstrated increases in plasma polyphenol levels and antioxidant capacity following their meal. The inclusion of red sorghum pasta in the diet resulted in a significant increase in SOD activity and a decrease in biomarkers of oxidative stress, including protein carbonyls. This evidence supports the notion that polyphenols derived from red sorghum are not only absorbed by the body but also exhibit biological activity, making them a noteworthy dietary component. Comparative analyses with wheat pasta further underscore the advantageous properties of red sorghum pasta as an effective strategy for alleviating oxidative stress, particularly for at-risk populations such as individuals with metabolic syndrome [25].

Sorghum, cultivated in various European countries, including Austria and Poland, is a rich source of antioxidant compounds, particularly phenolic acids and flavonoids. Notably, red sorghum exhibits the highest scavenging activities for ABTS and DPPH radicals. Whole-grain flour, which consists of 100% extraction, retains greater levels of antioxidant activity and bioactive compounds compared to refined flour. For instance, sorghum flour sourced from Poland can contain as much as 2850 mg/kg of phenolic acids. Consequently, it is advisable to prioritize the consumption of whole grains that include bran layers rather than refined flour products. This insight has significant industrial implications, indicating that sorghum can serve as a functional food with high antioxidant capacity, contributing to the reduction in dietary oxidative stress and aligning with growing consumer trends favoring whole grains [26,27].

Recent research into the antioxidant properties of sorghum underscores the grain’s abundance of beneficial compounds, including phenolic acids, flavonoids, carotenoids, and phytoalexins. The specific compounds present in sorghum can vary depending on its genotype and the processing methods employed. With advancements in metabolomics, it has become essential not only to identify the primary classes of antioxidants but also to determine their precise chemical structures. Notably, recent findings have uncovered formononetin and glycitein as novel compounds, enhancing our understanding of sorghum while facilitating the classification of different sources of antioxidants, metal chelating agents, and free radical scavengers [10]. The discovery of new isoflavones in sorghum carries significant implications for health, particularly in relation to inflammation and oxidative stress in living organisms; however, further mechanistic research is required to clarify the interactions and individual roles of these compounds.

Polyphenols derived from sorghum not only exhibit antioxidant properties but also have a significant impact on gut microbiota, particularly during periods of oxidative stress-related inflammation. A study conducted on animals with induced colitis demonstrated that diets enriched with sorghum bran effectively alleviated dysbiosis, a condition characterized by an imbalance in gut microbial diversity. More specifically, the polyphenol-rich diet modified the composition of the gut microbiota, restoring bacterial diversity by increasing populations of beneficial genera such as *Lactobacillus* and *Firmicutes*, while concurrently reducing the prevalence of bacteria associated with colitis. These findings suggest a link between the antioxidant properties of sorghum and the regulation of gut microbiome composition, ultimately leading to a reduction in oxidative stress-related inflammation [28].

### 3.2. Metabolic Health Benefits

The impact of sorghum consumption on the glycemic response has gained attention as a possible regulator of blood glucose levels. Several studies investigating European-grown sorghum types have determined that genotype plays a significant role in influencing the glycemic properties of foods, such as brown sorghum used for bread (low Glycemic Index, GI) versus bronze and white genotypes (medium GI). This evidence highlights the necessity of considering genotype-dependent effects on postprandial glycemic properties, which is applicable to other European varieties. Studies confirm that brown sorghum bread results in significantly lower glucose curves over a three-hour postprandial period [29].

Current reviews and meta-analyses suggest that various whole-grain sorghum products from Europe lead to a significantly lower three-hour area under the curve (AUC) for glycemic response compared to control products, such as refined wheat [29,30]. Notable glycemic benefits have been observed in several formulations, including bread, pasta, and porridge made with sorghum. However, the effects of long-term and habitual consumption of these sorghum-based foods on glycemic responses in European populations remain to be thoroughly investigated. The lower blood glucose levels associated with sorghum are linked to its low starch digestion and absorption, which have been shown to aid in the control of glycemia and the prevention of, e.g., obesity [30].

Intervention studies using animal models have shown that supplementation with fermented sorghum improves glycemic control. Across various experiments employing different doses of fermented sorghum alongside high-fat diets, medium- and high-dose supplementation significantly reduced postprandial glycemic responses by lowering both peak blood glucose levels and the total area under the curve during oral glucose tolerance tests. The resulting glycemic parameters were comparable to those observed with metformin. Moreover, supplementation with fermented sorghum was associated with an increased abundance of Bacteroidetes, accompanied by reductions in Firmicutes and Proteobacteria populations. The fermentation process modifies the structures of starches and proteins, thereby enhancing the bioavailability of phenolic compounds naturally present in sorghum. These phenolic compounds play a key role in boosting antioxidant activity, improving oral glucose tolerance, lowering blood pressure, and regulating carbohydrate and glucose absorption [31].

The phenolic acid content found in the outer layer of sorghum, particularly ferulic acid, *p*-coumaric acid, and caffeic acid, may play a significant role in its glycemic benefits. Research indicates that these phenolic acids can inhibit α-amylase and α-glucosidase in the digestive tract, thereby reducing carbohydrate and glucose metabolism in the small intestine. Additionally, these acids may enhance antioxidant capacity, which further aids in the regulation of blood sugar levels [32]. Despite these promising findings, more research is needed to better understand how these phenolic compounds affect glycemic status and how well they are absorbed. Although current evidence points to potential benefits, a clearer picture of sorghum’s influence on glycemic response is still required. Future studies should include long-term, large-scale, and well-controlled trials using European sorghum genotypes, assessing not only glycemic control but also broader metabolic markers such as insulin sensitivity and effects on gut microbiota. In addition, randomized controlled trials and meta-analyses are needed to determine how genotype, environmental conditions, and processing methods influence glycemic responses [30].

### 3.3. Weight and Inflammation Management, and Gut Microbiota Modulation

Weight control is essential in the fight against obesity, which is linked to various adverse health outcomes and remains a primary focus in research related to sorghum-based treatments. Randomized controlled trials (RCTs) have demonstrated that daily consumption of extruded sorghum significantly reduces body fat, waist circumference, and body weight in overweight individuals, particularly when compared to control groups consuming wheat or to baseline measurements. Notably, the reduction in body fat percentage was significantly more pronounced for those consuming sorghum (−2.97%) compared to wheat (−0.16%) [33]. These findings suggest that sorghum may offer valuable benefits for dietary therapy within a European context, particularly for the prevention of chronic diseases [34]. Nevertheless, as these studies have utilized non-European or unspecified sorghum genotypes and were conducted outside of Europe, further research is warranted on sorghum varieties produced in Europe to validate these results also in this continent. The table below (Table 1) lists the most important chemical compounds found in sorghum and their potential positive impact on the human body.

Sorghum is especially rich in unique polyphenols (particularly 3-deoxyanthocyanidins), which are rarely found in other cereals. These compounds give sorghum strong antioxidant and anti-inflammatory properties and may help prevent certain diseases, making the grain useful for functional foods and nutraceuticals. Some of sorghum’s health benefits may also come from its high content of resistant starch and dietary fiber, which are present in whole-grain sorghum grown in Europe [35,36]. These components enhance feelings of fullness and reduce subsequent caloric intake, thereby helping to prevent weight gain and facilitate weight loss. Intervention studies reveal that consuming sorghum-based foods leads to a stronger subjective sense of satiety compared to wheat-based alternatives. Moreover, sorghum consumption stimulates the release of appetite-regulating hormones, such as glucagon-like peptide-1, gastric inhibitory peptide, and peptide-tyrosine-tyrosine, which contribute to slower gastric emptying and improved appetite control. Collectively, these characteristics position sorghum as a promising food option for the prevention and management of obesity [35].

Another significant health aspect of sorghum related to weight management is its slow starch digestibility. Sorghum reduces the postprandial glycemic response due to its resistance to starch breakdown, contributing to more steady energy provision. This reduces rapid glucose surges, often associated with excessive caloric intake [36].

Sorghum consumption has also been shown to alter gut microbiota, leading to benefits in metabolic health in people who are overweight and obese. For example, several species (Prevotella sp. CAG:873 and Turicibacter) increased, while some microbial genera associated with obesity (*Clostridium* sensu stricto 1, *Dorea*, and *Odoribacter*) decreased in these individuals after consuming sorghum [34]. Moreover, sorghum dietary fiber, along with polyphenols, has also been reported to increase the populations of beneficial microbial groups, such as *Bifidobacterium*, *Lactobacillus*, *Roseburia*, and *Prevotella*. They have shown potential to improve energy metabolism and decrease inflammation [37]. All these factors suggest that it is crucial to identify which specific microbial groups are affected by European sorghum genotypes. Understanding these genotype–microbiota interactions could clarify how plant genetic variation influences gut microbial composition and metabolic health. Such insights may help determine whether European varieties promote beneficial microbes linked to improved energy metabolism and reduced inflammation. Ultimately, this knowledge could guide breeding and dietary strategies aimed at enhancing the nutritional and functional value of sorghum.

Research suggests that reducing uremic toxins, such as *p*-cresyl sulfate and indoxyl sulfate, can significantly alleviate inflammation, particularly for overweight and obese individuals who are prone to chronic inflammation and metabolic imbalances [35]. These compounds are byproducts of gut microbial metabolism. *p*-cresyl sulfate is mainly formed during the fermentation of aromatic amino acids such as tyrosine and phenylalanine, while indoxyl sulfate results from the breakdown of tryptophan. After being produced in the gut, they enter the bloodstream and are normally removed by the kidneys. However, when their levels become too high, they can contribute to systemic inflammation, oxidative stress, and endothelial dysfunction [35,37].

Therefore, approaches that modify gut microbiota activity or limit the buildup of these uremic toxins may help improve metabolic health and lower the risk of obesity-related complications. Recent studies show that whole-grain sorghum foods can positively influence the gut microbiota. For instance, one study examining a synbiotic sorghum meal in hemodialysis patients found that, compared with a maize-based control meal, the synbiotic sorghum meal led to significantly greater reductions in both *p*-cresyl sulfate (*p* < 0.05) and indoxyl sulfate (*p* < 0.05) [35,37].

### 3.4. Cardiovascular Health Promotion

The diverse range of polyphenolic compounds present in sorghum grains plays a significant role in promoting cardiovascular health through their free radical scavenging and antioxidant properties [38]. These benefits are vital for preventing or slowing the progression of atherosclerotic plaque formation and for mitigating the risk of other heart diseases. Additionally, sorghum can help reduce oxidative stress resulting from endothelial dysfunction and alleviate the low-grade inflammation commonly associated with early-stage cardiovascular issues. In fact, intervention studies have demonstrated that consuming sorghum-based foods can lead to lower levels of serum interleukin-6 and *C*-reactive protein, both of which are indicators of inflammation [34].

Sorghum features a distinctive nutritional profile that includes dietary fiber, plant sterols, and a low lipid content. Research indicates that plant sterols can effectively lower plasma cholesterol levels, as they are not readily absorbed by the intestinal walls, thereby helping to prevent cholesterol absorption. The cholesterol-lowering effects of sorghum can be further enhanced by its fiber content. Fiber binds to bile acids in the intestines, resulting in their excretion through stool, which in turn stimulates the production of bile acids from cholesterol in the liver. Furthermore, fiber inhibits the absorption of triglycerides by interfering with the activity of lipoprotein lipase (LPL) [38].

While sorghum has a low total lipid content (3–5%), it is primarily composed of beneficial unsaturated fatty acids, particularly oleic and linoleic acids [38]. The dietary intake of these fatty acids has been linked to improved blood lipid profiles, including lower total and LDL cholesterol and a slight increase in HDL cholesterol. These changes support better vascular health and may reduce the risk of hyperlipidemia, heart attack, and stroke [34].

Research also indicates that sorghum may help lower body fat percentage, waist circumference, and overall body weight—key factors in cardiovascular health. In one study, overweight individuals who consumed an extruded sorghum product showed better improvements in body composition and adiposity than those who consumed wheat. Sorghum may therefore help reduce cardiovascular risk factors related to central adiposity, since lowering abdominal fat is associated with a reduced risk of hypertension, coronary artery disease, and dyslipidemia [34].

Other studies explored the antihypertensive role of sorghum-derived bioactive peptides. Bioactive peptides can inhibit the ACE activity and prevent the formation of angiotensin II from angiotensin I. This action can decrease vasoconstriction and regulate high blood pressure. In fact, sorghum protein isolates present ACE inhibitory activity and lower the blood pressure. Furthermore, this biological activity is processing-dependent, and it has been demonstrated that germination (sprouted grains) exhibits lower antihypertensive activity compared to cooked or roasted sorghum, although it displays more antioxidant activity [39].

Resistant starch, dietary fiber, and polyphenols may work synergistically to promote cardiovascular health. These components contribute to feelings of satiety, influence energy intake, and can modify the composition of intestinal microbiota, which plays a crucial role in cardiometabolic health. For example, a study involving healthy participants demonstrated that sorghum resulted in significant changes in gut microbiota compared to a rice control group, specifically decreasing the counts of *Ruminococcus* while increasing the counts of *Bifidobacterium*. This suggests a potential microbiome-mediated mechanism through which sorghum may offer cardiovascular benefits, such as reduced inflammation and an improved lipid profile [34,36].

It would be interesting to further investigate and discuss the potential health benefits of sorghum in patients with established conditions, such as celiac disease or chronic kidney disease. Sorghum is naturally gluten-free, which is particularly important for individuals with celiac disease, allowing it to be safely incorporated into their diet. Additionally, exploring potential differences in sorghum’s effects between men and women, or across different age groups, could provide more nuanced insights. Sorghum grain has a very low uric acid content, with a maximum limit of 100 mg/kg specified by food standards, and is considered a low-purine food, which is beneficial for those managing gout. Previous studies have also shown that sorghum contains resistant starch, supporting glycemic control. In line with this, our team is planning studies to evaluate these effects in relevant patient populations. These properties highlight sorghum’s promise as a functional food in the dietary management of chronic diseases.

## 4. Research Collaboration Visualization of Sorghum in Healthcare

### 4.1. International Collaboration Network

**Figure 2:**
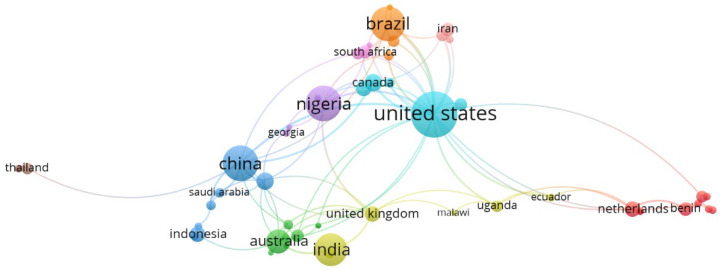
The bibliometric map obtained using VOSviewer software version 1.6.16 has shown the network visualization of countries involved in sorghum–healthcare research, based on the Scopus database.

Figure 2 presents the network visualization of countries involved in sorghum–healthcare research. Each node represents a country, with node size proportional to the number of publications and link thickness indicating the strength of international collab.

The United States emerges as the dominant hub, exhibiting extensive co-authorship links with China, Brazil, Nigeria, India, Australia, and the United Kingdom. This central position highlights the pivotal role of U.S.-based institutions in advancing multidisciplinary research on sorghum, ranging from plant biochemistry to nutritional and medical sciences. China and Australia form a tightly connected cluster, reflecting their collaboration in agricultural and nutritional studies involving sorghum as a functional food crop. Similarly, Brazil and Nigeria demonstrate strong co-authorship ties with the United States and other regions, indicating the typical South–North collaboration dynamics observed in plant-based and health-related research [17].

Peripheral nodes such as Iran, the Netherlands, and Thailand represent smaller yet specialized contributors. These countries often focus on niche areas, such as phytochemical extraction or ethnopharmacological studies, contributing to the diversity of research perspectives. The appearance of African and Asian countries—e.g., Uganda, Malawi, and Indonesia—illustrates the growing involvement of developing economies where sorghum serves as both a staple crop and a traditional medicinal resource [17,40,41,42].

Overall, the network suggests a globally interconnected research community, with cross-continental collaborations emphasizing food security, bioactive compounds, and the health-promoting potential of sorghum.

### 4.2. Keyword Co-Occurrence Network

**Figure 3:**
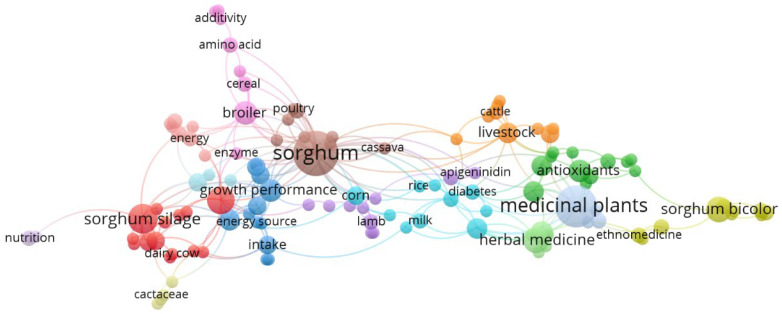
The bibliometric map obtained using VOSviewer software version 1.6.16 has shown the keyword co-occurrence network for the query “sorghum” AND (medicine OR healthcare), based on the Scopus database.

Figure 3 below illustrates the keyword co-occurrence network for the query “sorghum” AND (medicine OR healthcare). Each node represents a keyword, where larger nodes indicate higher frequency of occurrence, and the color grouping represents clusters of related topics.

Several distinct thematic clusters can be identified:

- **Cluster 1 (Red)**—Focused on nutritional and animal feed research, including terms such as “sorghum silage”, “growth performance”, “broiler”, “energy source”, and “nutrition”. These keywords highlight investigations into the use of sorghum in livestock feeding systems and its implications for animal health and productivity [11,43].
- **Cluster 2 (Green)**—Represents the pharmacological and medicinal dimension, featuring terms such as “medicinal plants”, “herbal medicine”, “antioxidants”, and “diabetes”. This cluster suggests an increasing interest in the biochemical composition and therapeutic potential of sorghum extracts, particularly phenolic compounds with antioxidant and antidiabetic properties [44,45].
- **Cluster 3 (Yellow/Blue)**—Integrates ethnopharmacological and biochemical research, containing keywords such as “apigeninidin”, “ethnomedicine”, and “sorghum bicolor”. These terms suggest studies exploring traditional medicinal uses and the molecular characterization of active components.
- **Cluster 4 (Orange)**—Links livestock and agricultural systems through terms like “cattle”, and “livestock”, representing the cross-section of agronomic and health-related studies.

Collectively, these clusters depict a multidimensional research field in which *sorghum* serves not only as a food and feed crop but also as a bioactive resource with applications in nutrition, medicine, and preventive healthcare.

### 4.3. Discussion

The bibliometric findings reveal that research on sorghum related to medicine and healthcare is interdisciplinary and globally distributed. The prominence of the United States, China, and Brazil as central nodes demonstrates the scientific and funding capacities of these nations in driving agricultural biotechnology and functional food studies [46]. Concurrently, active participation by Nigeria, India, and other developing countries underscores the significance of sorghum as a traditional medicinal and dietary crop in tropical and subtropical regions.

The integration of agricultural and biomedical research themes is evident from the co-occurrence of terms linking animal nutrition, antioxidant activity, and herbal medicine. Such overlap aligns with current global trends toward functional foods and nutraceuticals, where plant-derived compounds bridge the gap between nutrition and healthcare [42].

Moreover, the inclusion of keywords such as “diabetes”, “antioxidants”, and “herbal medicine” suggests a growing biomedical interest in sorghum-derived phenolics, particularly 3-deoxyanthocyanidins, such as apigeninidin, which exhibit anti-inflammatory and antioxidant effects relevant to chronic disease management [47,48,49]. These insights highlight sorghum’s potential to contribute to preventive medicine and functional food innovation, in line with global sustainable health strategies.

The collaboration patterns shown in Figure 1 further indicate that international cooperation is a key driver of innovation in this domain. Research partnerships between high-income and low- to middle-income countries are essential for leveraging local biodiversity knowledge and facilitating technology transfer. For example, joint projects between the United States and African nations often focus on improving crop resilience and exploring indigenous uses of sorghum for health-related purposes [50].

The adoption of an inclusive threshold (minimum occurrence = 1) allowed for a comprehensive mapping of both dominant and emerging contributors. While this approach increases network complexity, it provides a holistic overview of the field’s developmental stage. The dispersed keyword structure implies that the field is still fragmented yet evolving, suggesting opportunities for integration between agricultural science, phytochemistry, and medical research.

From a methodological standpoint, the bibliometric mapping demonstrates how visualization tools, such as VOSviewer, can effectively identify research clusters, knowledge gaps, and patterns of collaboration. The observed thematic convergence toward nutrition and health confirms that sorghum is transitioning from being primarily an agricultural commodity to a recognized component in health-promoting and therapeutic research frameworks [51].

## 5. Conclusions

A growing body of evidence demonstrates that sorghum exerts significant benefits on human health, particularly regarding glycemic regulation, antioxidant defense, body composition, and gut microbiota balance. Sorghum-based foods generally possess a low to moderate glycemic index, contributing to stable postprandial glucose levels and enhanced satiety, especially when consumed as whole-grain or bran-rich products. Clinical studies suggest that a daily intake of approximately 100 g of processed or extruded sorghum may reduce body fat and improve gastrointestinal health. In the context of Europe’s high consumption of processed foods and the widespread deficiency of dietary fiber and resistant starch, sorghum flour emerges as a promising ingredient for the prevention and management of obesity and metabolic syndrome. Moreover, its gluten-free nature provides a safe and palatable alternative for individuals with celiac disease or gluten sensitivity, expanding its relevance in functional food development.

Bibliometric and literature analyses reveal a dynamic and rapidly expanding research field. Global collaboration networks highlight strong North–South partnerships, while keyword mapping indicates increasing interdisciplinarity between agriculture, biochemistry, and health sciences. To advance the field, future research should focus on clinical validation, comprehensive characterization of bioactive compounds, and translational approaches that bridge laboratory findings with dietary applications. Nevertheless, while this review provides a comprehensive overview of sorghum’s health-promoting properties, several limitations should be considered. First, the narrative review approach, although systematic in its database search and critical content analysis, may be subject to selection bias, as it relies on the authors’ judgment in prioritizing studies for inclusion. The literature search was limited to peer-reviewed articles published in English between September and November 2025, which may have excluded relevant studies in other languages or recently published research outside this timeframe. Additionally, many of the primary studies included are either in vitro or animal-based, limiting the direct translation of findings to humans. Human clinical trials are relatively few, often with small sample sizes or short intervention durations, which restricts generalizability. Variability in sorghum genotypes, cultivation conditions, and processing methods further complicates comparisons across studies. Finally, interactions between sorghum bioactives and other dietary components or medications remain underexplored. Future research, particularly well-designed, long-term human studies, is needed to address these gaps and fully clarify the potential of sorghum as a functional food.

Further priorities include assessing nutrient bioavailability and biological activity across sorghum cultivars, as well as refining processing methods to preserve or enhance health-promoting properties. Robust human intervention studies, particularly within European populations, are essential to substantiate current findings. Additionally, local breeding programs should aim to improve both agronomic performance and nutritional quality. Ultimately, coordinated efforts by public and private sectors are needed to promote sorghum’s integration into European food systems. This review highlights sorghum’s potential as a sustainable, health-promoting cereal that aligns nutritional innovation with the prevention of chronic diseases and the advancement of global health.

## Figures and Tables

**Table 1:** The most important chemical compounds in sorghum and their potential positive impact on the human health.

Compound/Class	Type/ Category	Major Function in Plant	Potential Positive Impact on Human Health	Additional Notes	Sample References
**Phenolic acids** (e.g., ferulic acid, caffeic acid, *p*-coumaric acid)	Polyphenols/Atioxdants	Defense against UV and pathogens	Strong antioxidant activity; anti-inflammatory; may help reduce oxidative stress and risk of chronic diseases (CVD, diabetes, cancer)	Common in pericarp and bran; especially high in pigmented sorghum varieties	[20,21,27]
**Flavonoids** (e.g., apigenin, luteolin, naringenin)	Polyphenols	UV protection, pigmentation	Antioxidant and anti-inflammatory; potential neuroprotective and anticancer effects	Concentrated in the outer layers of the grain	[26,27,28]
**Tannins** (condensed tannins/proanthocyanidins)	Polyphenols	Pest deterrent	Antioxidant, antimicrobial, may help modulate glucose metabolism and gut health	High in brown and red sorghums; reduce protein digestibility if consumed excessively	[21,26,28]
**Phytosterols** (e.g., campesterol, stigmasterol, β-sitosterol)	Lipid compounds	Membrane stabilization	Cholesterol-lowering effects; support heart health	Found mainly in sorghum oil	[19,26,32]
**Dietary fiber** (soluble and insoluble)	Carbohydrate complex	Structural component	Improves digestion, supports gut microbiota, aids glycemic control and satiety	Whole-grain sorghum is rich in insoluble fiber	[7,9,11]
**Resistant starch**	Carbohydrate	Energy storage	Lowers postprandial glucose, improves gut health via fermentation to SCFAs	Increases with certain processing methods	[9,11]
**Tocopherols** **and Tocotrienols** (Vit. E compounds)	Lipid-soluble antioxidants	Protect lipids in plant tissues	Antioxidant and anti-aging effects; protect against oxidative stress	Found in germ and bran	[10,11,15]
**Peptides** **and amino acids** (bioactive proteins)	Proteins	Storage and defense	Antioxidant, antihypertensive, and antimicrobial properties (after enzymatic hydrolysis)	Released during digestion or fermentation	[8,10,15]

## Data Availability

This manuscript does not report research data generation.
